# Clinical and Radiographic Outcomes of Cementless Total Hip Arthroplasty for Osteoarthritis Secondary to Developmental Dysplasia of the Hip: A Retrospective Study with Minimum 12-Month Follow-Up

**DOI:** 10.3390/jfmk11030251

**Published:** 2026-06-26

**Authors:** Francesco Liuzza, Pierfrancesco Pirri, Gianluca Serra, Andrea Aureli, Fernando De Maio, Pasquale Farsetti, Giuseppe Rovere

**Affiliations:** Section of Orthopaedics and Traumatology, Department of Clinical Science and Translational Medicine, University of Rome “Tor Vergata”, 00133 Rome, Italy

**Keywords:** hip reconstruction, cementless implants, acetabular augmentation, femoral head autograft, Crowe classification, Hartofilakidis classification, soft-tissue release, limb length restoration, implant fixation, survivorship analysis

## Abstract

**Background:** Developmental dysplasia of the hip (DDH) is one of the leading causes of secondary hip osteoarthritis and frequently results in severe anatomical alterations that make total hip arthroplasty (THA) technically demanding. Restoration of hip biomechanics, limb length, and joint stability remains challenging, particularly in patients with moderate-to-severe dysplasia. **Objective:** To evaluate the clinical and radiographic outcomes of cementless total hip arthroplasty combined with soft-tissue balancing, with or without acetabular reconstruction using autologous femoral head graft, in patients affected by osteoarthritis secondary to DDH. **Methods:** A retrospective single-center case series was conducted on eight female patients (mean age 53.9 ± 14.6 years; range 33–80 years) who underwent primary cementless THA for DDH-related osteoarthritis between 2019 and 2025. Clinical outcomes were assessed using the Harris Hip Score (HHS), Western Ontario and McMaster Universities Osteoarthritis Index (WOMAC), and Short Form-36 (SF-36). Radiographic evaluation included implant positioning, osteolysis, heterotopic ossification, bone graft incorporation, and leg-length discrepancy. Data normality was assessed using the Shapiro–Wilk test. Preoperative and postoperative outcomes were compared using paired Student’s *t*-test and confirmed with the Wilcoxon signed-rank test. Implant survival and revision-free status were recorded throughout the follow-up period. **Results:** At a minimum follow-up of 12 months (range 12 months–6 years), significant improvements were observed in all clinical outcome measures. Mean HHS increased from 49.3 ± 2.5 preoperatively to 90.4 ± 2.7 postoperatively (*p* < 0.001), while mean WOMAC decreased from 53.5 ± 5.6 to 7.4 ± 3.3 (*p* < 0.001). Mean SF-36 improved from 47.2 ± 3.8 to 89.9 ± 3.2 (*p* < 0.001). Wilcoxon analysis confirmed the statistical significance of these findings (all *p* = 0.0078). Radiographic assessment demonstrated satisfactory implant positioning and stable fixation in all patients, with no evidence of osteolysis or implant loosening. Minor complications included one intraoperative periprosthetic femoral fracture treated successfully with cerclage wiring, two cases of Brooker grade I–II heterotopic ossification, and one case of minimal graft resorption without clinical consequences. No revision procedures were recorded during follow-up, and implant survival was 100%. **Conclusions:** Cementless THA combined with selective soft-tissue balancing provides excellent clinical, functional, and radiographic outcomes in patients with osteoarthritis secondary to DDH. This approach significantly improves hip function and quality of life while ensuring stable implant fixation, low complication rates, and excellent mid-term implant survival.

## 1. Introduction

Developmental dysplasia of the hip (DDH) encompasses a spectrum of congenital and developmental abnormalities affecting the acetabulum, proximal femur, and surrounding soft tissues, resulting in abnormal hip joint morphology and altered biomechanics [1,2,3,4]. The incidence of DDH has been reported to range from 1 to 10 cases per 1000 live births, although considerable variations exist among different populations and geographic regions [1,2,3,4]. The condition occurs more frequently in females and is associated with several recognized risk factors, including breech presentation, positive family history, oligohydramnios, and ligamentous laxity influenced by maternal and fetal hormonal factors [5,6,7]. DDH may present unilaterally in approximately 60–75% of cases and bilaterally in the remaining patients [1,2,3,4]. Despite advances in neonatal screening and early treatment strategies, DDH remains one of the most common causes of secondary hip osteoarthritis and a major indication for total hip arthroplasty (THA) in young and middle-aged adults [8].

The pathophysiology of DDH is characterized by abnormal development of both the acetabular and femoral components of the hip joint. The dysplastic acetabulum is typically shallow, vertically oriented, and deficient in superolateral coverage, leading to insufficient containment of the femoral head [1,2,3,4]. On the femoral side, increased anteversion, valgus neck-shaft angle, rotational deformities, and narrowing of the femoral canal are frequently encountered [9]. These anatomical abnormalities alter the physiological distribution of joint forces and increase contact pressures across the articular cartilage, resulting in progressive degeneration and early onset osteoarthritis [8]. Furthermore, chronic subluxation or dislocation of the femoral head may lead to adaptive shortening of periarticular soft tissues, muscle imbalance, gait alterations, and limb-length discrepancy, all of which contribute to functional impairment and complicate surgical reconstruction in adulthood [10,11].

Early diagnosis and treatment are essential to prevent irreversible degenerative changes. In infancy and early childhood, treatment strategies are aimed at achieving stable reduction of the femoral head within the acetabulum and promoting normal joint development. Non-operative management includes observation, serial clinical and radiographic monitoring, and the use of orthotic devices such as the Pavlik harness or abduction braces, depending on the patient’s age and severity of dysplasia [5,6,7]. When conservative treatment fails or diagnosis is delayed, surgical intervention may be required. Various reconstructive procedures have been described, including open reduction, femoral osteotomy, Salter osteotomy, Pemberton osteotomy, Dega osteotomy, Chiari osteotomy, and other pelvic realignment procedures designed to improve acetabular coverage and restore hip stability during skeletal growth [8].

Despite advances in pediatric treatment, a substantial proportion of patients eventually develop symptomatic osteoarthritis requiring joint replacement. Consequently, THA has become the treatment of choice for patients with end-stage degenerative changes secondary to DDH [9]. However, THA in dysplastic hips remains one of the most technically demanding procedures in adult reconstructive orthopaedics. Significant acetabular bone deficiency, proximal femoral deformity, altered femoral anteversion, soft-tissue contractures, previous corrective surgeries, and limb-length discrepancy frequently require individualized surgical planning and complex intraoperative decision-making [9,10,11].

Several classification systems have been developed to describe the severity of dysplasia and facilitate preoperative planning. The Crowe classification is widely used to quantify the degree of proximal migration of the femoral head, whereas the Hartofilakidis classification provides a more detailed anatomical description of the relationship between the femoral head and acetabulum [9,10,11]. These systems are valuable tools for estimating surgical complexity, determining the need for acetabular reconstruction, and evaluating the possible indication for adjunctive procedures such as femoral shortening osteotomy.

Current evidence consistently demonstrates that THA provides substantial pain relief and significant improvements in function and quality of life in patients with DDH. Nevertheless, several aspects of surgical management remain controversial, including the optimal reconstruction of acetabular bone defects, restoration of the native hip center of rotation, management of limb-length discrepancy, and the role of subtrochanteric shortening osteotomy in severe dysplasia [9,10,11]. Previous studies have highlighted the importance of restoring physiological hip biomechanics in order to improve implant survival and functional outcomes. More recently, advances in cementless implant design, acetabular reconstruction techniques, and soft-tissue balancing strategies have expanded the indications for anatomical reconstruction, even in complex dysplastic hips [9,10,11].

Although encouraging results have been reported, the available literature remains heterogeneous with regard to patient selection, surgical techniques, and outcome assessment. Additional evidence is therefore required to better define the effectiveness of contemporary cementless THA combined with selective soft-tissue balancing and acetabular reconstruction in patients affected by osteoarthritis secondary to DDH.

The aim of the present study was to evaluate the clinical and radiographic outcomes of cementless total hip arthroplasty performed for osteoarthritis secondary to developmental dysplasia of the hip at a minimum follow-up of 12 months.

We hypothesized that cementless THA combined with selective soft-tissue balancing and, when necessary, acetabular reconstruction using autologous femoral head graft would provide significant improvements in hip function, pain relief, quality of life, and radiographic parameters, while maintaining a low complication rate and excellent implant survival.

## 2. Materials and Methods

### 2.1. Study Design and Setting

This retrospective single-center case series was conducted at the Department of Orthopaedics and Traumatology, University of Rome “Tor Vergata”, Rome, Italy. The study was performed in accordance with the ethical principles of the Declaration of Helsinki. All patients provided written informed consent for the use of their anonymized clinical and radiographic data for research purposes.

Given the retrospective observational design and the absence of any intervention beyond standard clinical practice, prospective clinical trial registration was not applicable. Furthermore, no a priori sample size calculation was performed because of the relatively low incidence of patients affected by advanced osteoarthritis secondary to developmental dysplasia of the hip (DDH) treated at a single tertiary referral center. Therefore, all consecutive patients meeting the eligibility criteria during the study period were included in the analysis.

### 2.2. Patient Selection and Participants’ Characteristics

A retrospective review of institutional medical records was performed to identify patients affected by advanced osteoarthritis secondary to DDH who underwent primary cementless total hip arthroplasty (THA) between January 2019 and January 2025.

Patients were included if they had a diagnosis of osteoarthritis secondary to DDH, underwent primary cementless THA at our institution, had complete clinical and radiographic documentation, availability of preoperative and postoperative Harris Hip Score (HHS), Western Ontario and McMaster Universities Osteoarthritis Index (WOMAC), and Short Form-36 (SF-36) questionnaires, and a minimum follow-up of 12 months.

Patients were excluded if THA had been performed for causes other than DDH, including primary osteoarthritis, avascular necrosis, femoral neck fracture, inflammatory arthritis, or post-traumatic arthritis. Additional exclusion criteria included revision arthroplasty, conversion procedures, active or previous periprosthetic joint infection, severe neurological or neuromuscular disorders affecting gait, severe systemic diseases limiting reliable follow-up, incomplete clinical records, and patients treated with subtrochanteric shortening osteotomy.

A total of eight female patients fulfilled the inclusion criteria and were included in the final analysis. The mean age at surgery was 53.9 ± 14.6 years (range 33–80 years), and the follow-up period ranged from 12 months to 6 years. Three patients had undergone previous corrective procedures during childhood, including Chiari pelvic osteotomy and distalization of the greater trochanter. Demographic, clinical, radiographic, and functional data were collected for all patients. These included age, sex, body mass index (BMI), previous surgical procedures, side involved, follow-up duration, and quality-of-life assessment.

### 2.3. Preoperative Clinical and Radiographic Evaluation

All patients underwent a comprehensive preoperative clinical and radiographic evaluation aimed at defining a patient-specific surgical strategy.

Standard radiographic assessment included anteroposterior pelvis radiographs obtained with 10–15° internal rotation, axial hip radiographs, and full-length standing radiographs of both lower limbs. When required, computed tomography (CT) scans with three-dimensional reconstruction were performed to improve visualization of complex anatomical deformities.

**Figure 1:**
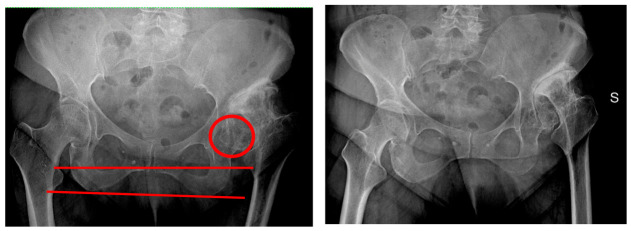
Preoperative anteroposterior pelvis radiograph demonstrating acetabular dysplasia and superior migration of the femoral head, used for surgical planning.

**Figure 2:**
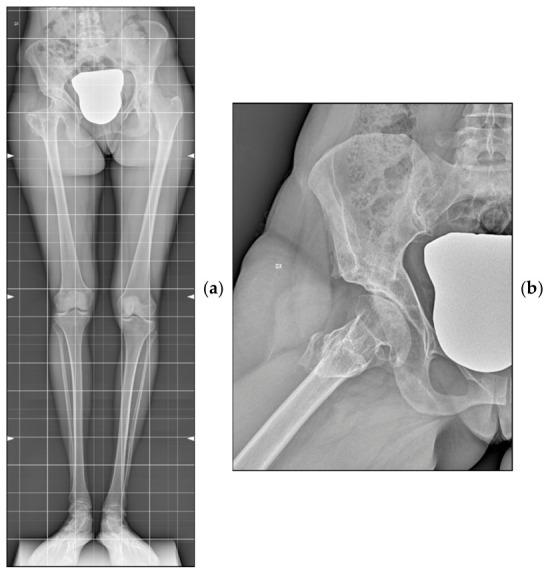
(**a**) Full-length standing radiograph demonstrating lower limb length discrepancy (arrow); (**b**) axial hip radiograph showing proximal femoral morphology and increased femoral anteversion.

**Figure 3:**
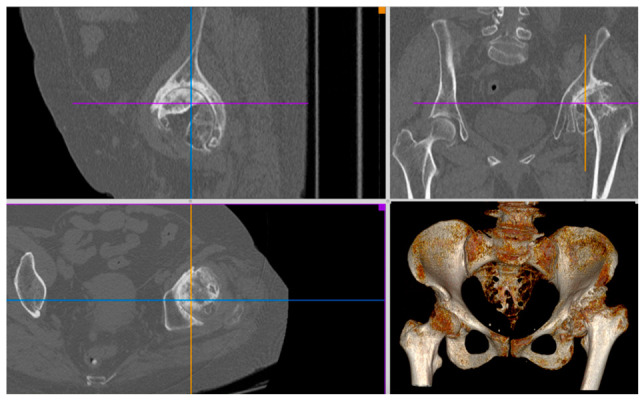
Three-dimensional CT reconstruction showing acetabular bone deficiency, true acetabulum location, and femoral deformity used for preoperative planning.

Figure 1 shows a representative preoperative anteroposterior pelvis radiograph used for surgical planning. This examination allowed assessment of acetabular morphology, superior migration of the femoral head, joint congruency, and acetabular bone stock. Full-length standing radiographs were used to evaluate lower-limb alignment and leg-length discrepancy (LLD), while axial hip radiographs provided information regarding proximal femoral morphology and rotational deformities (Figure 2a,b). In selected cases presenting severe deformities, CT scans with three-dimensional reconstruction were obtained to facilitate identification of the true acetabulum, assessment of acetabular bone deficiencies, evaluation of femoral anteversion, and planning of implant positioning (Figure 3).

The following parameters were systematically evaluated during preoperative planning:

- Identification of the true acetabulum;
- Determination of the anatomical hip center of rotation;
- Acetabular bone stock and bone defects;
- Femoral morphology and canal dimensions;
- Femoral anteversion;
- Femoral offset;
- Limb-length discrepancy;
- Bone quality according to the Dorr classification;
- Severity of dysplasia according to the Crowe and Hartofilakidis classifications.

The primary objectives of preoperative planning were restoration of the native hip center of rotation, optimization of hip biomechanics, correction of limb-length discrepancy, restoration of femoral offset, and achievement of stable implant fixation.

### 2.4. Surgical Technique

All surgical procedures were performed by the same senior surgeon using a standardized posterolateral (Moore) approach.

**Figure 4:**
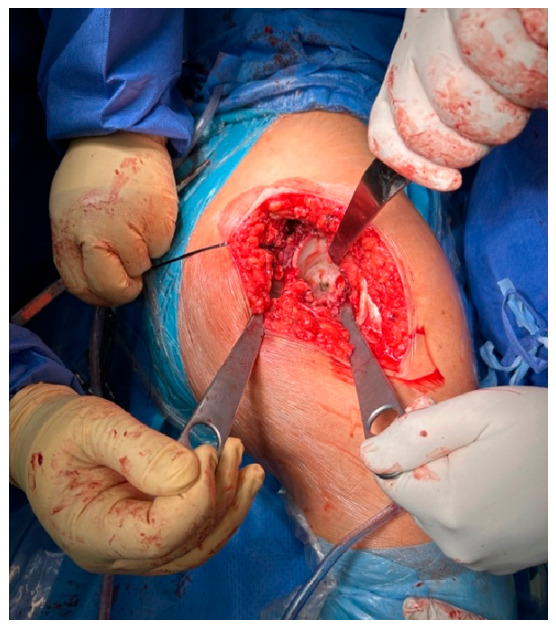
Intraoperative identification of the true acetabulum following femoral neck osteotomy.

Following femoral neck osteotomy, the true acetabulum was identified and progressively prepared. This surgical step is illustrated in Figure 4, which demonstrates intraoperative identification of the native acetabulum and preparation of the acetabular bed for anatomical cup positioning.

**Figure 5:**
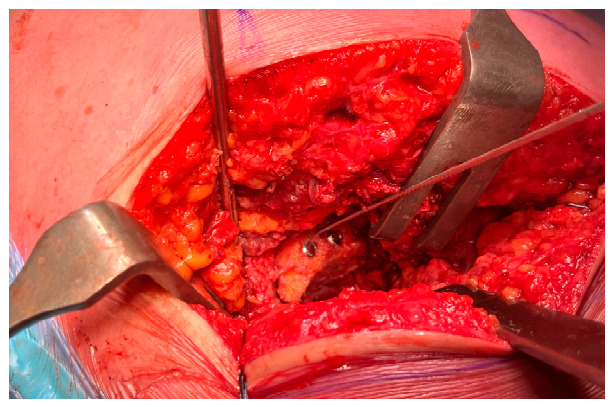
Structural autologous femoral head graft fixed with cancellous screws to improve acetabular coverage.

The acetabular component was implanted at the level of the true acetabulum with a target inclination of approximately 40–45° and anteversion of 10–15°. A cementless porous titanium acetabular component (Delta TT^®^, LimaCorporate, San Daniele del Friuli, Italy) was used in all patients. The bearing surface consisted of a ceramic femoral head articulating with a highly cross-linked polyethylene liner. Component positioning and fixation were performed according to the manufacturer’s recommendations and individualized anatomical requirements. In cases presenting significant acetabular bone deficiency, structural autologous femoral head grafts were harvested, shaped, and fixed with cancellous screws to improve acetabular coverage and facilitate restoration of the anatomical hip center (Figure 5).

**Figure 6:**
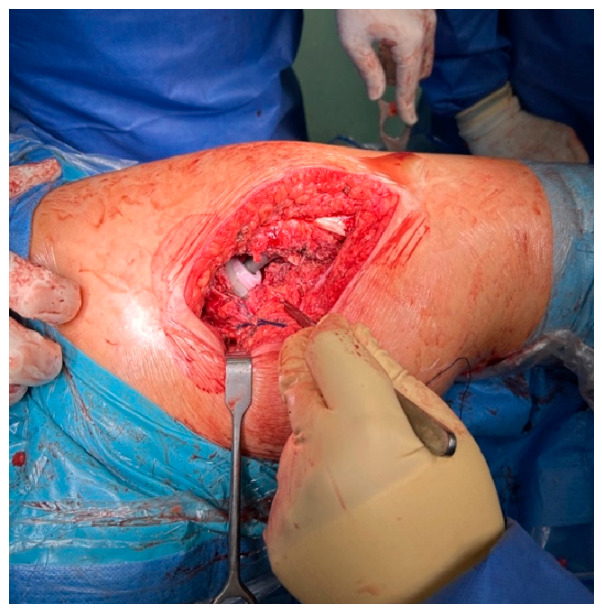
Final implantation of a cementless modular femoral stem with restoration of hip biomechanics.

Femoral preparation was performed using sequential broaches until satisfactory metaphyseal fixation was achieved. Trial components were inserted to evaluate hip stability, offset restoration, limb length, and soft-tissue tension before implantation of the definitive cementless components. Figure 6 illustrates the final cementless modular femoral stem (Lima Modulus stem) after implantation.

Selective soft-tissue balancing was performed when necessary and included release of contracted structures such as the adductor magnus tendon, iliopsoas tendon, and, in selected cases, portions of the gluteus maximus. Soft-tissue release was undertaken only when persistent soft-tissue tightness following trial reduction prevented stable hip reduction, adequate restoration of limb length, satisfactory soft-tissue balance, or appropriate joint stability. The extent of release was individualized according to intraoperative findings in order to facilitate reduction while minimizing the risk of excessive limb lengthening and neurological complications.

Complete restoration of limb-length equality was not systematically pursued in order to minimize the risk of neurological complications associated with excessive lengthening.

### 2.5. Clinical and Functional Evaluation

Clinical and functional outcomes were assessed preoperatively and at final follow-up using validated outcome measures.

Hip function was evaluated using the Harris Hip Score (HHS). Pain, stiffness, and functional limitations were assessed using the Western Ontario and McMaster Universities Osteoarthritis Index (WOMAC). WOMAC results are reported as raw scores, with higher values indicating greater pain, stiffness, and functional impairment. Health-related quality of life was evaluated using the Short Form-36 (SF-36).

### 2.6. Radiographic Evaluation

Postoperative radiographic assessment was performed using standardized anteroposterior pelvis and hip radiographs obtained during routine follow-up visits. Radiographic evaluation was independently performed by two senior orthopaedic surgeons experienced in hip arthroplasty who were not involved in the collection of clinical outcome scores. Radiographs were reviewed immediately after surgery, at 6 weeks, at 12 months, and annually thereafter until the latest available follow-up. The evaluators assessed implant positioning and alignment, evidence of implant loosening, osteolysis according to Gruen zones, heterotopic ossification according to the Brooker classification, acetabular graft incorporation and possible graft resorption, restoration of limb length, and overall implant stability and fixation. Any discrepancies in radiographic interpretation were resolved by consensus. In addition, preoperative dysplasia severity according to the Crowe and Hartofilakidis classifications, femoral anteversion, femoral offset, limb-length discrepancy, and bone quality according to the Dorr classification were recorded and subsequently analyzed.

### 2.7. Statistical Analysis

Statistical analysis was performed using SPSS software version 25.0 (IBM Corp., Armonk, NY, USA).

Continuous variables were expressed as mean ± standard deviation (SD), minimum values, and maximum values. Categorical variables were reported as absolute frequencies and percentages.

Normality of data distribution was assessed using the Shapiro–Wilk test, whereas homogeneity of variances was evaluated using Levene’s test. Parametric data were analyzed using paired Student’s *t*-tests. Given the limited sample size, Wilcoxon signed-rank tests were additionally performed as sensitivity analyses to confirm the robustness of the results.

A two-tailed *p*-value < 0.05 was considered statistically significant.

## 3. Results

### 3.1. Patients’ Characteristics

A total of eight female patients who underwent primary cementless total hip arthroplasty (THA) for osteoarthritis secondary to developmental dysplasia of the hip (DDH) were included in the study. Baseline demographic, anthropometric, and radiographic characteristics of the study population are summarized in Table 1.

The mean age at surgery was 53.9 ± 14.6 years (range, 33–80 years). All patients completed the scheduled clinical and radiographic follow-up evaluations and had complete preoperative and postoperative functional outcome data available. The follow-up period ranged from a minimum of 12 months to a maximum of 6 years.

Three patients (37.5%) had previously undergone corrective surgical procedures during childhood, including Chiari pelvic osteotomy and distalization of the greater trochanter. Preoperative assessment demonstrated varying degrees of dysplasia according to both the Crowe and Hartofilakidis classification systems. Additional radiographic parameters, including femoral anteversion, femoral offset, bone quality according to the Dorr classification, and limb-length discrepancy, were recorded during preoperative planning and are reported in Table 1.

No patients were lost to follow-up, and all were available for final clinical, functional, and radiographic assessment.

### 3.2. Clinical Outcomes

Clinical outcomes were evaluated using the Harris Hip Score (HHS), Western Ontario and McMaster Universities Osteoarthritis Index (WOMAC), and Short Form-36 (SF-36). Statistical analyses were performed using paired Student’s *t*-test and confirmed by Wilcoxon signed-rank test because of the limited sample size.

**Figure 7:**
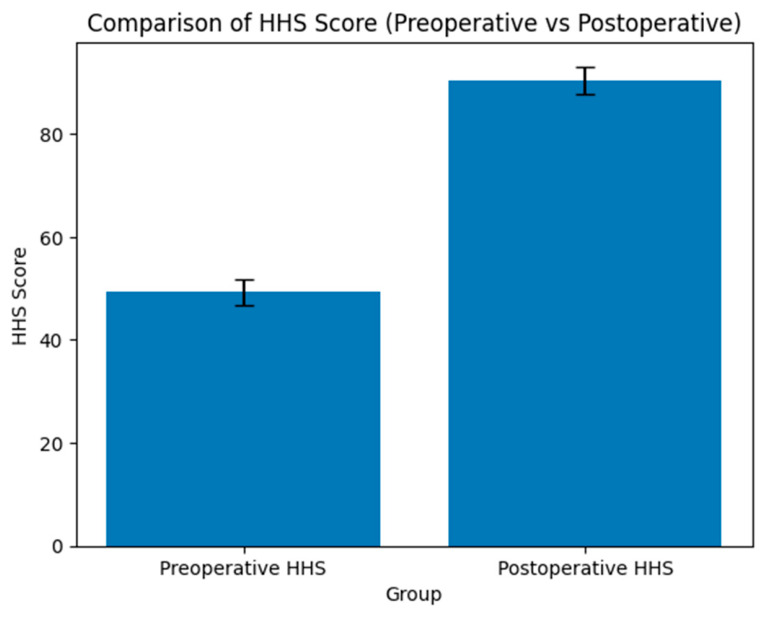
Comparison of pre- and post-operative HHS in patients undergoing total hip arthroplasty. Values are expressed as a means ± standard deviation (SD).

A significant improvement in hip function was observed following surgery. The mean HHS increased from 49.3 ± 2.5 preoperatively to 90.4 ± 2.7 at the latest follow-up (Table 2, Figure 7). This improvement was highly significant according to both the paired Student’s *t*-test (*p* < 0.001) and the Wilcoxon signed-rank test (*p* = 0.0078), indicating substantial recovery of hip mobility, pain relief, and restoration of daily activities.

**Figure 8:**
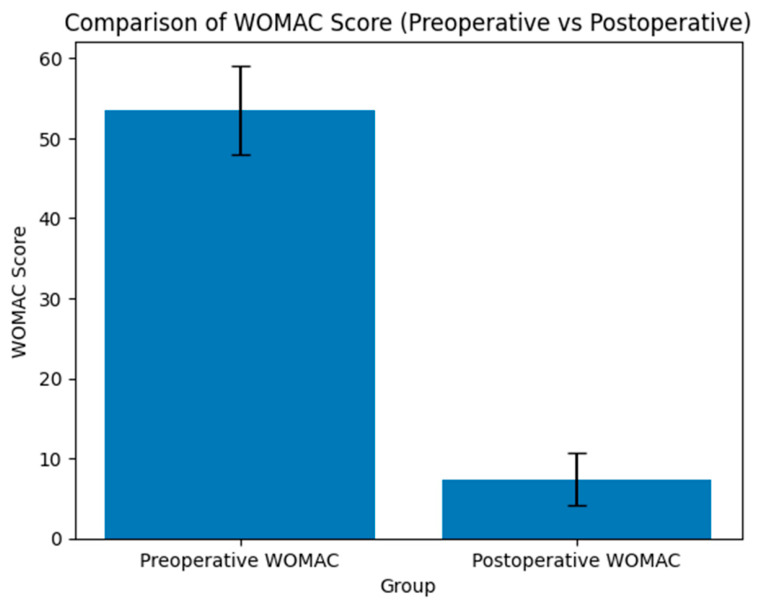
Comparison of pre- and post-operative total WOMAC score in patients undergoing total hip arthroplasty. The values are expressed as the Means ± SD. The reduction in the WOMAC score in the post-operative period is statistically significant (Student’s *t*-test for paired samples *p* < 0.001; Wilcoxon test *p* = 0.0078.).

Similarly, the total WOMAC score demonstrated a marked reduction from 53.5 ± 5.6 before surgery to 7.4 ± 3.3 at final follow-up (Table 3, Figure 8). The observed reduction was statistically significant according to both Student’s *t*-test (*p* < 0.001) and Wilcoxon analysis (*p* = 0.0078), reflecting substantial improvements in pain, stiffness, and functional capacity following THA.

Subgroup analysis of WOMAC domains revealed statistically significant improvements in both pain and functional limitation. The pain score decreased from 6.25 ± 1.39 to 0.88 ± 0.35 (*p* < 0.001), while the functional limitation score improved from 22.38 ± 3.70 to 5.38 ± 3.07 (*p* < 0.001). Conversely, the stiffness domain showed only a modest reduction from 2.38 ± 0.52 to 2.00 ± 0.93, which did not reach statistical significance (Student’s *t*-test *p* = 0.401; Wilcoxon *p* = 0.625) (Table 4).

**Figure 9:**
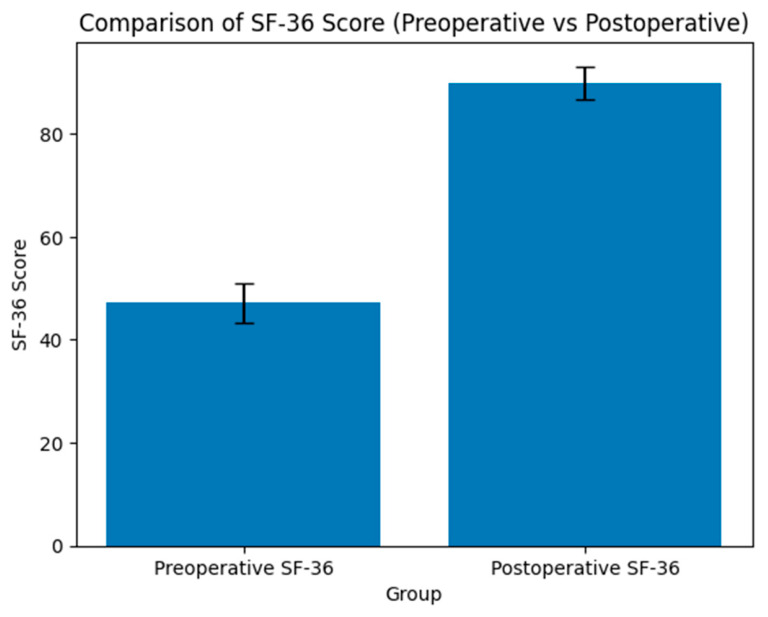
Comparison of total pre- and post-operative SF-36 score in patients undergoing total hip arthroplasty. The values are expressed as the mean ± SD. The improvement in the total SF-36 score in the post-operative period was statistically significant (Student’s *t*-test for paired samples *p* = 2.8 × 10^−8^; Wilcoxon test *p* = 0.0078).

Quality-of-life assessment demonstrated a substantial improvement following surgery. The mean SF-36 score increased from 47.2 ± 3.8 preoperatively to 89.9 ± 3.2 at final follow-up (Table 5, Figure 9). This improvement was statistically significant according to both Student’s *t*-test for paired samples *p* < 0.001; Wilcoxon test *p* = 0.0078), indicating marked enhancement in physical health, emotional well-being, social functioning, and overall health perception.

Overall, all clinical outcome measures demonstrated statistically and clinically significant improvements following cementless THA. The combination of substantial HHS improvement, marked WOMAC reduction, and enhanced SF-36 scores indicates successful restoration of hip function, effective pain relief, and a significant positive impact on patient quality of life.

### 3.3. Radiographic Outcomes

Radiographic analysis focused on implant position, stability, leg length discrepancy, and the presence of osteolysis or heterotopic ossifications.

**Figure 10:**
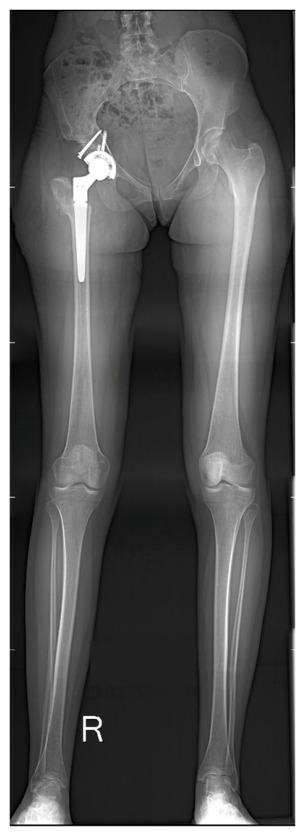
Patient undergoing THA, autologous bone grafting from femoral head and soft tissue balancing following distalization of the greater trochanter. A well-tolerated residual LLD of 1.7 cm is found at follow-up.

**Figure 11:**
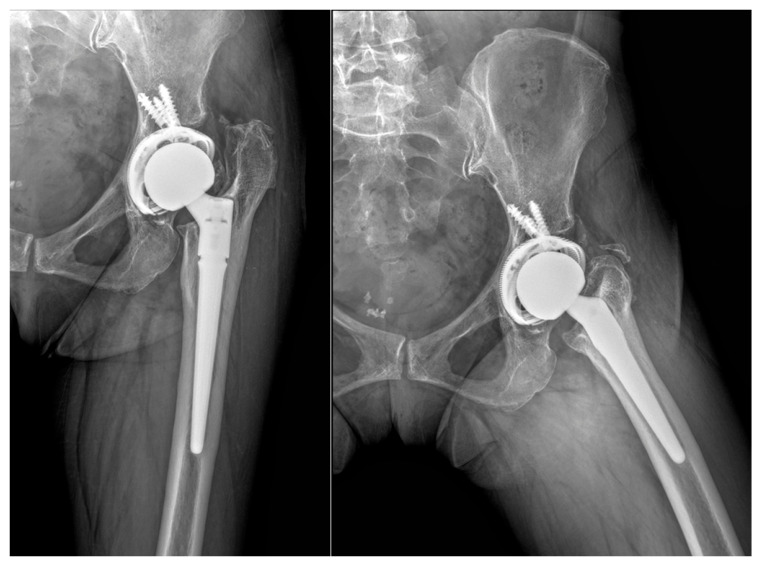
Heterotopic ossification in patient without functional limitation and clinical symptoms.

- Implant Position and Stability
  ▪ All prosthetic components were correctly positioned, with satisfactory alignment of both acetabular and femoral components.
  ▪ Osteolysis: No radiographic signs of osteolysis were observed in any of the Gruen zones, indicating absence of bone resorption or implant loosening during follow-up.
  ▪ Implant Stability: No cases of radiographic loosening or mechanical failure were detected, confirming stable fixation of all components.
- Leg Length Discrepancy (LLD)
  ▪ A partial correction of preoperative LLD was achieved in several patients.
  ▪ Residual LLD, when present, was consistently less than 1.7 cm and was clinically well tolerated, without functional impairment or need for further intervention (Figure 10).
- Heterotopic Ossifications
  ▪ Grade I-II (Brooker classification): Observed in 2 patients, without associated functional limitation or clinical symptoms (Figure 11).
  ▪ No higher-grade heterotopic ossifications were detected.

Overall, radiographic findings confirmed satisfactory implant positioning, stable fixation, and absence of clinically relevant complications throughout the follow-up period.

The radiographic outcomes for implant position and stability, leg-length discrepancy and heterotopic ossifications are summarized in Table 6.

### 3.4. Postoperative Complications

No major complications were observed during the follow-up period. Specifically, no neurological or vascular complications, periprosthetic joint infections, implant loosening, or radiographic signs of osteolysis were detected. Additionally, no patients reported significant residual hip pain.

One intraoperative periprosthetic femoral fracture occurred during stem implantation and was successfully managed with cerclage wiring, without compromising implant stability or clinical outcome.

Two patients developed heterotopic ossification classified as Brooker grade II; however, these findings were not associated with functional limitations and did not require further treatment.

In one patient, minimal resorption of the autologous bone graft used for acetabular reconstruction was observed. This finding remained radiographically limited and did not affect implant stability or acetabular coverage.

Overall, the complication rate was low and consistent with previously reported outcomes in THA performed for severe DDH.

The postoperative complications and their corresponding details are summarized in Table 7.

**Figure 12:**
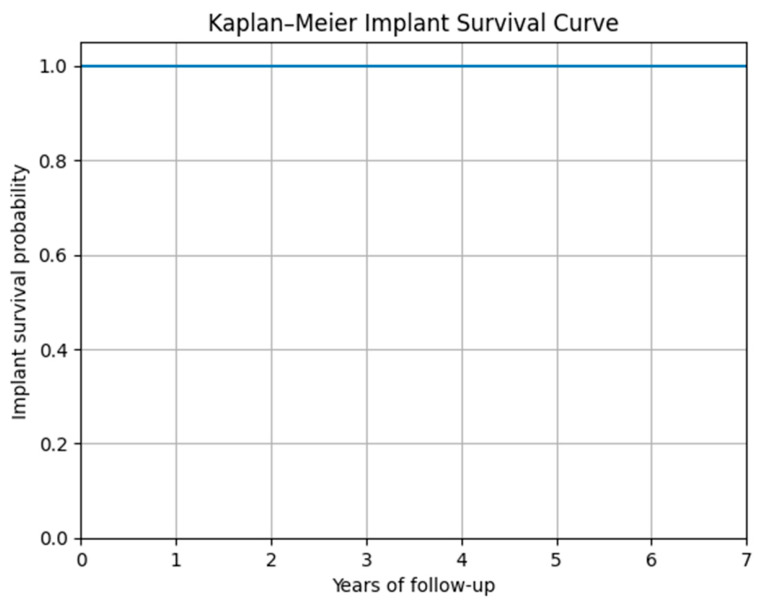
Kaplan–Meier implant survival curve in patients undergoing total hip arthroplasty for severe developmental dysplasia of the hip.

Implant survival analysis, performed using the Kaplan–Meier method with revision for any cause as the endpoint, demonstrated a 100% survival rate at the available follow-up (Figure 12).

No implant failures or revision procedures were recorded during the observation period, confirming the good mid-term reliability of the prosthetic components.

No squeaking phenomena were detected during clinical evaluation.

Overall, the clinical, functional, and quality-of-life outcomes indicate that total hip arthroplasty in patients with severe DDH provides significant improvement in joint function, substantial pain relief, and marked enhancement of quality of life, with excellent implant survival at the available follow-up.

## 4. Discussion

Developmental dysplasia of the hip (DDH) remains one of the most challenging conditions encountered in adult reconstructive orthopaedics. Anatomical abnormalities such as acetabular bone deficiency, proximal femoral deformity, increased femoral anteversion, soft-tissue contractures, and limb-length discrepancy significantly complicate total hip arthroplasty (THA) and may affect both functional recovery and implant survival. Consequently, meticulous preoperative planning and individualized surgical strategies are essential to restore hip biomechanics and achieve satisfactory clinical outcomes [11,12]. The principal finding of the present study is that cementless THA combined with selective soft-tissue balancing and, when necessary, acetabular reconstruction using autologous femoral head graft resulted in substantial functional improvement, excellent radiographic outcomes, and a low complication rate in patients with osteoarthritis secondary to DDH.

The clinical results observed in the present series demonstrated significant improvements in all evaluated outcome measures. Harris Hip Score, WOMAC, and SF-36 scores improved markedly from preoperative values, indicating restoration of joint function, reduction of pain, and improvement in overall quality of life. These findings are consistent with previous reports demonstrating that THA is an effective treatment for end-stage osteoarthritis secondary to DDH and can provide functional outcomes comparable to those achieved in primary osteoarthritis when adequate reconstruction is obtained [13,14,15]. The significant increase in HHS observed in our cohort reflects improved mobility, enhanced weight-bearing capacity, and restoration of daily activities. Similarly, the marked reduction in WOMAC scores confirms substantial relief of pain and disability, which remain the primary goals of arthroplasty surgery in this patient population.

Quality-of-life assessment using the SF-36 questionnaire demonstrated a significant postoperative improvement across multiple domains. In addition to improvements in physical function, patients reported better emotional well-being, social participation, and overall perception of health status. These findings suggest that successful reconstruction of dysplastic hips provides benefits extending beyond pain relief and joint function alone. Previous studies have emphasized the importance of patient-reported outcome measures in evaluating the effectiveness of THA in DDH, as these patients often experience severe functional limitations for many years before surgery [15]. The present findings further support the role of modern cementless THA as an effective strategy for improving both objective and subjective outcomes.

One of the key objectives of THA in dysplastic hips is restoration of normal hip biomechanics. Positioning the acetabular component within the true acetabulum allows reconstruction of the native center of rotation and restoration of the abductor lever arm, thereby improving gait mechanics and reducing residual limping. Several authors have demonstrated that anatomical reconstruction provides more physiological load distribution and may contribute to improved implant longevity [11,16,17,18,19,20]. In the present study, careful preoperative planning combined with intraoperative identification of the true acetabulum allowed satisfactory restoration of hip anatomy in all patients. Radiographic analysis confirmed stable component positioning and absence of implant loosening throughout the follow-up period.

Management of acetabular bone deficiency remains a critical aspect of THA in DDH. Structural autologous femoral head grafting was used selectively in patients with insufficient acetabular coverage, allowing anatomical cup positioning and restoration of bone stock [20,21,22,23,24,25]. The majority of grafts demonstrated satisfactory incorporation, while only one patient presented minimal graft resorption that did not compromise implant stability or acetabular coverage. These findings are consistent with previous reports supporting the use of structural autografts in dysplastic hips [26,27]. Furthermore, biomechanical studies have demonstrated that biological grafts and porous metal augments may improve acetabular component stability and optimize load transmission across the bone–implant interface [28,29]. Therefore, acetabular reconstruction remains an important tool when anatomical cup placement cannot be achieved using native bone alone.

Correction of limb-length discrepancy (LLD) represents another important challenge during THA in patients with DDH. Excessive lengthening may increase the risk of neurological complications, particularly sciatic nerve injury, whereas inadequate correction may compromise gait and patient satisfaction. In the present series, a substantial reduction of preoperative LLD was achieved while maintaining neurological safety. Residual discrepancies remained below 2 cm and were well tolerated clinically. These findings support previous recommendations suggesting that functional restoration and soft-tissue balance should be prioritized over complete anatomical equalization when excessive lengthening may place neurovascular structures at risk [20,21,22].

Management of the proximal femur remains equally important in dysplastic hips. Increased femoral anteversion, deformity of the proximal femur, and narrowing of the femoral canal may occasionally require additional reconstructive procedures. Although subtrochanteric shortening osteotomy is widely recognized as a valuable option in selected cases of severe dysplasia, particularly Crowe III and IV deformities, it was not required in the present series. Careful preoperative planning, selective soft-tissue balancing, and restoration of the anatomical hip center allowed stable reduction in all patients included in the study. While femoral shortening osteotomy remains an important surgical option, our findings suggest that satisfactory reconstruction may be achieved without routine use of this procedure in appropriately selected patients [23,24,25].

The complication profile observed in this study was favorable. No neurological injuries, vascular complications, infections, implant failures, dislocations, or revision procedures occurred during follow-up. One intraoperative periprosthetic fracture was successfully managed with cerclage wiring without affecting implant stability or clinical outcome. In addition, two patients developed low-grade heterotopic ossification without functional consequences. These findings are comparable with previously published series reporting low complication rates following modern cementless THA in DDH patients [15]. The absence of revision surgery resulted in an implant survival rate of 100% at the latest available follow-up, further supporting the reliability of the surgical strategy adopted in this cohort.

The present study has several limitations that should be acknowledged. In addition, outcome assessors were not blinded to the surgical procedure or preoperative clinical status, which may have introduced a potential source of observational bias. First, its retrospective design introduces an inherent risk of selection bias. Second, the relatively small sample size limits statistical power and restricts the generalizability of the findings. Furthermore, the study population consisted exclusively of female patients. Although this reflects the well-established epidemiology of developmental dysplasia of the hip, which predominantly affects women, it may limit the generalizability of the findings to male patients. However, DDH requiring THA remains a relatively uncommon and heterogeneous condition, particularly when strict inclusion criteria are applied. Third, the absence of a control group prevents direct comparison with alternative reconstructive techniques or different implant designs. Finally, all procedures were performed by a single experienced surgeon, which ensured technical consistency but may limit external validity. Despite these limitations, the study provides clinically relevant information regarding the effectiveness of cementless THA combined with selective soft-tissue balancing and acetabular reconstruction in patients with osteoarthritis secondary to DDH.

## 5. Conclusions

The present study demonstrated that cementless total hip arthroplasty combined with selective soft-tissue balancing, and acetabular reconstruction using autologous femoral head graft when required, is a safe and effective treatment for osteoarthritis secondary to developmental dysplasia of the hip.

This surgical strategy resulted in significant improvements in hip function, pain relief, and health-related quality of life, as demonstrated by the marked postoperative increases in HHS and SF-36 scores and the substantial reduction in WOMAC scores. Radiographic evaluation confirmed satisfactory restoration of hip biomechanics, stable implant fixation, successful acetabular reconstruction, and absence of implant loosening or osteolysis throughout follow-up.

Furthermore, the procedure was associated with a low complication rate and achieved 100% implant survival at the latest available follow-up. These findings support the use of contemporary cementless THA as a reliable reconstructive option for patients with DDH-related osteoarthritis, capable of restoring function, improving quality of life, and providing favorable radiographic outcomes.

Although larger prospective studies with longer follow-up are required to confirm these results, the present findings suggest that meticulous preoperative planning, anatomical reconstruction of the hip center, and selective soft-tissue balancing are key factors for achieving successful outcomes in this challenging patient population.

## Figures and Tables

**Table 1:** Demographic and preoperative characteristics of the study population (N = 8).

Variable	Value
Number of patients	8
Female sex	8 (100%)
Mean age (years)	53.9 ± 14.6
BMI (kg/m^2^)	27.3 ± 3.9
Follow-up	12 months–6 years
Previous corrective surgery	3 (37.5%)
Crowe classification	
Crowe I	0 (0%)
Crowe II	3 (37.5%)
Crowe III	4 (50.0%)
Crowe IV	1 (12.5%)
Hartofilakidis classification	
Type A (Dysplasia)	3 (37.5%)
Type B (Low Dislocation)	4 (50.0%)
Type C (High Dislocation)	1 (12.5%)
Dorr classification	
Type A	4 (50.0%)
Type B	3 (37.5%)
Type C	1 (12.5%)

**Abbreviations:** BMI, Body Mass Index.

**Table 2:** Comparison of preoperative and postoperative Harris Hip Score (HHS). Values are expressed as mean ± standard deviation (SD). Statistical significance was assessed using paired Student’s *t*-test and Wilcoxon signed-rank test.

Score	n	Mean ± Standard Deviation (SD)	*p*-Value (Student’s *t* Test)	*p*-Value (Wilcoxon)
Preoperative HHS	8	49.3 ± 2.5	*p* < 0.001	0.0078
Postoperative HHS	8	90.4 ± 2.7	*p* < 0.001	0.0078

**Table 3:** Comparison of preoperative and postoperative WOMAC scores. Values are expressed as mean ± SD. Statistical significance was assessed using paired Student’s *t*-test and Wilcoxon signed-rank test.

Score	n	Mean ± SD	*p*-Value (Student’s *t* Test)	*p*-Value (Wilcoxon)
Preoperative WOMAC	8	53.5 ± 5.6	*p* < 0.001	0.0078
Postoperative WOMAC	8	7.4 ± 3.3	*p* < 0.001	0.0078

**Table 4:** Comparison of preoperative and postoperative WOMAC subscale scores.

WOMAC Subscales	n	Preoperative (Mean ± SD)	Postoperative (Mean ± SD)	*p*-Value (Student’s *t* Test)	*p*-Value (Wilcoxon)
Pain	8	6.25 ± 1.39	0.88 ± 0.35	*p* < 0.001	0.0078
Stiffness	8	2.38 ± 0.52	2.00 ± 0.93	0.401	0.625
Functional limitation	8	22.38 ± 3.70	5.38 ± 3.07	*p* < 0.001	0.0078

**Table 5:** Comparison of preoperative and postoperative SF-36 scores. Values are expressed as mean ± SD. Statistical significance was assessed using paired Student’s *t*-test and Wilcoxon signed-rank test.

Score	n	Mean ± (SD)	*p*-Value (Student’s *t* Test)	*p*-Value (Wilcoxon)
Preoperative total SF-36	8	47.2 ± 3.8	*p* < 0.001	0.0078
Postoperative total SF-36	8	89.9 ± 3.2	*p* < 0.001	0.0078

**Table 6:** Summary of postoperative radiographic findings.

*Radiographic Parameters*	*Value*
Osteolysis (Gruen zones)	None
Leg Length Discrepancy	1.7 cm
Heterotopic Ossification (Grade I-II)	2 patients (25%)
Heterotopic Ossification (Grade III-IV)	None
Bone Graft without resorption	7 patients (87.5%)
Bone Graft with minimal resorption	1 patient (12.5%)
Implant Survival	100%

**Table 7:** Postoperative complications.

Type of Complication	Cases	Details
Major complications	0 (0%)	No neurological, vascular, or infectious complications observed
Intraoperative periprosthetic fracture	1 (12.5%)	Treated with cerclage wiring; no impact on implant stability or outcome
Heterotopic ossification (Brooker II)	2 (25%)	No functional limitation; no further treatment required
Bone graft resorption	1 (12.5%)	Minimal and radiographically limited; no effect on stability or coverage

## Data Availability

The datasets used and/or analyzed during the current study are available from the corresponding author on reasonable request.

## Abbreviations

The following abbreviations are used in this manuscript:

DDH	Developmental Dysplasia of the Hip
THA	Total Hip Arthroplasty
HHS	Harris Hip Score
WOMAC	Western Ontario and McMaster Universities Arthritis Index
SF-36	Short Form-36 Health Survey
LLD	Leg Length Discrepancy
SD	Standard Deviation
PJI	Periprosthetic Joint Infection
IRB	Institutional Review Board
ROM	Range of Motion
